# Prevention of Pneumonia

**Published:** 1884-05

**Authors:** 


### THE PREVENTION OF PNEUMONIA.

EVERY Winter, inflammation of the lungs destroys the lives of many persons who might have escaped if the preventive measures here advocated had been effectively practised at the proper time. In order to render the value of the latter more apparent, it is necessary to notice very briefly the influence of breathing, on the body and on the food required for its support. The capacity possessed by the living body to vitalize nutritive materials is perhaps its most wonderful physical endowment. Every step in this marvellous process requires the vivifying influence of oxygen. Without an abundant supply of this gas to the system through respiration, the food cannot be properly fitted to repair the wasting tissues; the body is therefore necessarily repaired by materials having a low degree of vitality. Again, as oxygen is the agent by which effete matters are reduced to those forms by which their complete removal from the system is facilitated, if it be not breathed in adequate amount, the body becomes clogged by its own waste.

As in chemical manipulations a definite quantity of an alkali is required to saturate an acid of given volume and strength, so in the vitalization of food, a definite quantity of the vital gas, oxygen, is required to enable the system to fully complete the vitalization of a given amount of food. A man of average weight requires about two pounds of solid food per diem, and very nearly the same weight of oxygen is absorbed into the blood from the respired air; therefore, we shall not be far from the truth when we assume that an atom of food requires to be acted upon in the living body by an atom of oxygen, in order that its vitalization may be effected.

The most important anatomical change occurring during the progress of pneumonia is the solidification of a larger or smaller part of one or both lungs, by the deposit in the terminal bronchial tubes and the air-cells, of a substance by which the spongy lungs are rendered almost as solid and impenetrable to air as bone. The access of the respired air to the solidified part being totally prevented, life is inevitably destroyed if a sufficiently large part of the lungs be invaded. This deposit succeeds the first or congestive stage; it occurs with great rapidity; an entire lobe of the lung may be rendered perfectly solid by the exudation from the blood of fully two pounds of solid matter in the short space of twelve hours, or even less. The rapidity with which the lungs become solidified, accounts for the promptly fatal results that often attend attacks of acute pneumonia. If recovery takes place, the foreign matter by which the lung tissue has been solidified is perfectly absorbed, and the recently diseased portion is found to be quite uninjured.

The only natural method by which the blood can be freed from the presence of waste matter is by its oxidation, the results being carbonic acid gas that escapes by the lungs, and certain materials that are eliminated chiefly by the kidneys. But when these impurities exist in the vital fluid in unusually large quantities, or if the respiratory capacity be inadequate, the natural internal crematory operations are a partial failure.

But nature cannot long tolerate the presence of such impurities in the vital fluid; if they cannot be eliminated by natural means, they must be got rid of by means involving diseased action; therefore, such material is frequently deposited in various parts of the body, the point of deposit being usually determined by some local disturbance or irritation. The liability of any person to attacks of acute pneumonia is determined chiefly by the presence or absence in his blood of the waste matter referred to, and by the condition of the respiratory power. If the blood be free from any abnormal amount of such waste matter, because his respiratory capacity is up to the full requirements of the system, no cold, however severe, is competent to originate the disease. But if the blood be charged with the matter, a very moderate irritation will determine an attack.

There can be no question but that high living and sedentary habits have a strong tendency to befoul the blood. The former renders effective respiration all the more necessary for the removal from the system of whatever nutritive matter has been taken, beyond the needs of the physical necessities, while the latter inevitably reduces the respiratory motions to the lowest point consistent with physical comfort.

These conditions are active predisposing causes of pneumonia. The disease is more fatal in the very young and in the aged :— The mortality for acute pneumonia seems to bear a direct ratio to the respiratory capacity; in young subjects the breathing powers have not been fully developed, while in the aged the respiratory volume has been diminished by the stiffening of the chest walls and of the lungs themselves by the senile changes incident to the decline of life. The most effective preventive measures that can be adopted against attacks of acute pneumonia is to keep the breathing up to the full requirements of the system.

A few minutes spent each day in simple but effective exercises adapted to expand the chest, will, in the course of a few weeks, render the lungs much more permeable to air. The volume of each respiration may thus be increased by two or even three cubic inches of air, but if we assume that the gain is one cubic inch, the aggregate increase in the volume of air breathed in the course of twenty-four hours would amount to about as many cubic feet.

